# Modelling the potential geographic distribution of triatomines infected by *Triatoma virus* in the southern cone of South America

**DOI:** 10.1186/s13071-015-0761-1

**Published:** 2015-03-12

**Authors:** Soledad Ceccarelli, Agustín Balsalobre, María Laura Susevich, María Gabriela Echeverria, David Eladio Gorla, Gerardo Aníbal Marti

**Affiliations:** Centro de Estudios Parasitológicos y de Vectores (CEPAVE-CCT-La Plata-CONICET – UNLP), Boulevard 120 e/61 y 62, 1900 La Plata, Argentina; Cátedra de Virología, Facultad de Ciencias Veterinarias, Universidad Nacional de La Plata, (CONICET), La Plata, Buenos Aires, Argentina; Centro Regional de Investigaciones Científicas y Transferencia Tecnológica (CRILAR - CONICET), La Rioja, Argentina

**Keywords:** Triatoma virus, Triatominae, Ecological Niche Modelling, MaxEnt, WorldClim, AVHRR imagery

## Abstract

**Background:**

*Triatoma virus* (TrV) is the only entomopathogenous virus identified in triatomines. We estimated the potential geographic distribution of triatomine species naturally infected by TrV, using remotely sensed and meteorological environmental variables, to predict new potential areas where triatomines infected with TrV may be found.

**Methods:**

Detection of TrV infection in samples was performed with RT-PCR. Ecological niche models (ENM) were constructed using the MaxEnt software. We used 42 environmental variables derived from remotely sensed imagery (AVHRR) and 19 bioclimatic variables (Bioclim). The MaxEnt Jackknife procedure was used to minimize the number of environmental variables that showed an influence on final models. The goodness of fit of the model predictions was evaluated by the mean area under the curve (AUC).

**Results:**

We obtained 37 samples of 7 species of triatomines naturally infected with TrV. Of the TrV positive samples, 32% were from sylvatic habitat, 46% came from peridomicile habitats and 22% from domicile habitats. Five of the seven infected species were found only in the sylvatic habitat, one species only in the domicile and only *Triatoma infestans* was found in the three habitats. The MaxEnt model estimated with the Bioclim dataset identified five environmental variables as best predictors: temperature annual range, mean diurnal range, mean temperature of coldest quarter, temperature seasonality and annual mean temperature. The model using the AVHRR dataset identified six environmental variables: minimum Land Surface Temperature (LST), minimum Middle Infrared Radiation (MIR), LST annual amplitude, MIR annual amplitude annual, LST variance and MIR variance. The potential geographic distribution of triatomine species infected by TrV coincides with the Chaco and the Monte ecoregions either modelled by AVHRR or Bioclim environmental datasets.

**Conclusions:**

Our results show that the conditions of the Dry Chaco ecoregion in Argentina are favourable for the infection of triatomine species with TrV, and open the possibility of its use as a potential agent for the biological control of peridomestic and/or sylvatic triatomine species. Results identify areas of potential occurrence that should be verified in the field.

**Electronic supplementary material:**

The online version of this article (doi:10.1186/s13071-015-0761-1) contains supplementary material, which is available to authorized users.

## Background

Chagas disease is an endemic zoonotic disease of the American continent that currently affects between 7 and 8 million people [1]. Around 1.6 million people are infected in Argentina and between 15 - 30% of them present cardiac injuries or irreversible lesions in other organs [2].

Although the main transmission route is the vectorial one in Latin American countries, the causative agent of this disease, *Trypanosoma cruzi*, can also be transmitted via congenital transmission, blood transfusion or organ transplantation from infected donors, or by eating food contaminated with triatomine faeces. Because of the global human migrations, Chagas disease is now installed in both large cities of Latin America and in non-endemic countries, especially in the USA and Spain [1].

*Trypanosoma cruzi* is the most studied intestinal microorganism associated with triatomines due to its sanitary importance. However, other microorganisms associated with triatomines were collected in field studies, as part of efforts to identify potential biological control agents in Argentina, e.g. the flagellated protozoan *Blastocrithidia triatomae,* the entomopathogenic fungi *Beauveria bassiana* and *Paecilomyces lilacinus;* and the pathogenic virus *Triatoma virus* (TrV) [3].

There are at least 14 families of pathogenic viruses of invertebrates that have been studied as biological control agents of insects, being *Dicistroviridae* one of the most important families [4]. In 2002, the International Committee in Taxonomy of Virus included the TrV within the *Dicistroviridae*, next to a small group of insect viruses (*Cripavirus*), being the cricket paralysis virus (CrPV) the species type and in the last few years has been proposed Triatovirus as a new genus [5,6]. *Triatoma virus* and *Solenopsis invicta virus-1* (SINV-1) are the only members of this family with public health importance, and there are also eight members which are pathogens of insect pests of crops that could be used as biological control agents [7,8].

*Triatoma virus* is the only entomopathogenic virus identified in triatomines and its structure consists of 9,010 nucleotide positive-sense ssRNA genome that is encapsulated by 60 copies of structural proteins VP2, VP4, VP3 and VP1 [9]. The viral particles are spherical (30 nanometres in diameter) and lack envelope. TrV replicates in the cells of the intestinal epithelium of triatomines, causing death of the infected individuals [10]. Pathogeny of the virus and transmission routes have been determined partially, indicating that coprophagy is one of the main routes of infection in laboratory colonies of triatomines [11,12], a behaviour that also facilitates the transmission of parasites and symbionts [13].

TrV was found and identified by Muscio *et al.* [14] and subsequently found in field collections of *Triatoma infestans, T. sordida, T. delpontei,* and *Psammolestes coreodes* in Argentina [3,15]. Several of these species along with *Rhodnius prolixus, T. pallidipenis, T. maculata, T. guasayana, T. rubrovaria, T. platensis, T. dimidiata* and *T. patagonica* were infected artificially by intrahaemocoelic or oral routes, and showed similar mortality [10,16]. TrV was recently found in an insectary in Brazil in *Rhodnius neglectus* and *Meccus longipennis* [16]. The ecology of TrV and its capability as a biological control agent of triatomines are currently under investigation. No study on its geographic distribution has ever been reported.

In general, Species Distribution Models (SDMs) estimate the relationship between species records at sites and the environmental and/or spatial characteristics of those sites [17]. Various methods have been used to predict species potential distribution using Ecological Niche Modelling (ENM) based on occurrence data points and environmental variables, showing generally good results [18]. ENM may be particularly significant in vector studies, suggesting potential areas of vector occurrence, and consequently identifying risk areas of pathogen transmission [19] and possibly their entomopathogens.

The aim of this work is to estimate the potential geographic distribution of triatomine species naturally infected by *Triatoma virus* (TrV), using remotely sensed and meteorological environmental variables. It is the first time that this kind of analysis has been carried out in relation to pathogens of triatomine species and predicting new potential areas where triatomines infected with TrV may be found.

## Methods

### Data collection

Samples of triatomine specimens were collected from August 2002 to August 2013 in rural areas of fifteen provinces of Argentina (Buenos Aires, Catamarca, Córdoba, Chaco, Formosa, La Rioja, Mendoza, Misiones, Neuquén, Salta, San Juan, San Luis, Santa Fe, Santiago del Estero and Tucumán) and in two departments of Bolivia (Santa Cruz and Cochabamba). Each field sample included all triatomines belonging to the same species collected in the same habitat. Each habitat was classified as domicile, peridomicile, and sylvatic environment. In the domiciles and peridomicile ones, the collection was carried out by active manual search by three members of our research staff that captured as many specimens as possible. In the sylvatic environment, the sample collection was carried out by active and passive search, the latter included Noireau traps [20] and light traps. About 15–20 Noireau traps were left overnight (in each field collection) baited with a mouse enclosed within a container with an external adhesive tape that would trap the triatomine that approached to feed. In addition, eight light traps were placed in sylvatic environments in La Rioja and Chaco provinces, every 100 meters (transect of about 800 m) during four hours (between 7 pm and 11 pm) for six consecutive nights in each field collection. The sampled domiciles had adobe walls, thatched roofs, and between two to four rooms. Some houses had brick walls and roofs of corrugated metal sheeting. Such a dwelling covered by a single roof defined the domicile area. The peridomestic environment consisted of places located within the area of human activity and included a wide array of structures such as sheep, goat, and pig corrals, chicken coops and store rooms. In the sylvatic environment, triatomines were collected strictly in the wild environment such as bird nests, mammal refuges, cactus, bromeliads and tree bark.

The collected insects were transported to the laboratory in sterile plastic containers with folded pieces of common paper inside, capped with a fine screen and were maintained at 28 ± 2°C, 60 ± 5% HR, and a photoperiod of 12:12 h (light:dark). Triatomines were identified according to [21]. Triatomine samples were geo-located using a handheld Garmin™ Legend GPS navigator.

Detection of TrV infection in faecal material samples (fresh samples) was performed with (RT-PCR) as described by [22]. When samples had < 20 specimens, they were analysed individually and in cases where the samples had > 20 specimens, analysis was performed using pools with a maximum of 10 specimens to detect and then confirm TrV presence by Transmission Electronic Microscope at 250 K magnification.

### Ecological niche modelling

#### Species input data

Samples from triatomines naturally infected by TrV were used as occurrence points for the ENM procedure. As TrV susceptibility and mortality of the different species of triatomine are similar [10,16] and the number of occurrence points of each species was small (<20), we grouped all species to obtain the MaxEnt input dataset, and therefore fulfilled the minimum sample size criterion of occurrence data to permit a robust ENM development [23]. Duplicate records were eliminated.

#### Environmental data

We used two different environmental datasets to characterize environmental variation: multi-temporal remotely sensed imagery and bioclimatic data. The study area was South America (12.67 North, −82.35 West and −56.55 South, −33.84 East).

##### a) Multi-temporal remotely sensed imagery

Images produced by the AVHRR (Advanced Very High Resolution Radiometer) on board of the NOAA (National Oceanic and Atmospheric Administration) meteorological satellite series were used. This sensor detects visible and infra-red radiation between 0.3 - 14 μm in five bands, from which bio-physical properties of the earth surface can be estimated. We used a monthly temporal series between 1982–2000, processed using the maximum value composite method [24] to reduce cloud contamination. The spatial resolution of the images was 8 × 8 km. The environmental information of the images included data on land surface temperature (LST), normalized difference vegetation index (NDVI), and middle infrared radiation (MIR), whose reflected component from a vegetation canopy is a function of the liquid water content of the canopy [25]. The monthly temporal series was analysed using the temporal decomposition of Fourier. Using this method, a time series can be expressed as a function of sine functions with different amplitudes and phases around a characteristic mean. It is therefore possible to extract information about annual, bi-annual cycles of vegetation, temperatures and other variables that characterize the natural environment [26]. For example, the phase of the annual cycle of the Fourier analysis describes the moment of the year when a sinusoidal curve reaches the highest value, and is related with the seasonality of a variable. In this case, the statistics describing each time series were amplitudes and phases of the annual, bi-annual and tri-annual cycles, the variance accounted for by each of these three cycles; and mean, minimum, maximum and variance values of the Fourier description of the original series. A total of 14 statistics from each environmental variable was considered to characterize each 8x8 km pixel from the study area with a total of 42 variables (see Table A2 in the Additional file 1). The Fourier products, kindly provided by the TALA group (Oxford University, UK), offer the most stable synoptic surfaces used for monitoring global scale environmental conditions of relevance to infectious disease mapping [27].

##### b) Bioclimatic data

The other environmental dataset consisted of bioclimatic variables characterizing climate during 1950–2000, drawn from the WorldClim dataset [28]. Variables included 19 bioclimatic statistics derived from monthly total precipitation, and monthly mean, minimum and maximum temperature, and altitude (see Table A1 in the Additional file 1). We used all variables at 5 arc-minute spatial resolutions (9 × 9 km approximately).

The WorldClim dataset is being widely used in studies on the geographic distribution of biological species. Its main weakness is the amount of data interpolation to obtain the geographic coverage of temperature and rainfall from meteorological ground stations whose density in the study area is low (see http://www.worldclim.org/methods). AVHRR dataset has been less used than the WorldClim, although it depends less on data interpolation as it is derived from remote sensing records.

To avoid the confounding effects of calibrating models in an overly dimensional environmental space, we used the Jackknife procedure in MaxEnt (see above) to reduce the number of environmental variables to only those that showed a substantial influence on the distribution of the TrV infection. The basic process was: 1) use all the variables (42 from AVHRR and 19 from WorldClim) to run each model, 2) check the Jacknife test results and omit the variable which has the most negative effect on the total gain (in other words, using the remaining variables would get more gain than including this variable) and those variables which do not have a normal response curve, 3) use the remaining variables to run the model and check the Jacknife test results to omit another variable. Finally, 4) repeat step 2) y 3) until all the remaining variables have a positive effect on the total gain.

#### Modelling

Ecological niche models (ENM) were constructed using the MaxEnt software (Version 3.3.3) [29], oriented towards modelling species distributions from presence-only species records with a predictive performance consistently competitive with the highest performing methods [30].

We used default parameters, except that we chose the option “random seed” and we used the subsample replicated run type with 100-fold with 70% of the records to train and 30% to test the model. We chose the average of the 100-fold output grids as the best hypothesis of potential range. We used 20,000 points of background to cover the range of environmental conditions in the modelled region [31]. Finally, we set the option to create response curves to show how predicted relative probability of occurrence depends on the value of each environmental variable.

The goodness of fit of the model predictions was evaluated by the mean area under the curve (AUC) of the Receiver Operating Characteristic curve, routinely calculated for each run by MaxEnt. The AUC varies from 0 to 1, with a value of 1 indicating a perfect prediction. An AUC threshold value of 0.80 was set to consider acceptable as a predictive model. Although the AUC goodness of fit has been criticized [32], it is very useful for identifying suitable areas of occurrence and comparing models of species distribution [30,33].

## Results

### Data collection

A total of 298 samples (293 collected in Argentina and 5 in Bolivia), including 5977 insects were collected, identified and used for the detection of TrV infection. The positive TrV samples were found mainly in the arid Chaco and Monte ecoregions in Argentina [34].

From these samples, we identified 10 triatomine species: *Panstrongylus guentheri*, *Ps. coreodes*, *T. breyeri*, *T. delpontei*, *T. eratyrusiformis*, *T. garciabesi*, *T. guasayana*, *T. infestans*, *T. platensis* and *T. sordida*, only three of which were not found infected by TrV (*P. guentheri, T. eratyrusiformis* and *T. garciabesi*). *Triatoma breyeri* has been found for the first time infected with TrV, increasing the number of susceptible species to 15. Five of the seven infected species were found only in the sylvatic habitat (*Ps. coreodes, T. breyeri, T. delpontei, T. guasayana and T. platensis*), one species only in the domicile (*T. sordida*) and only *T. infestans* was found in the three habitats (Table 1).

**Table 1:** **Number of samples by species and positivity to TrV infection collected in each habitat**

**Species**	**Total samples**	**Samples (−) TrV**			**Samples (+) TrV**		
		**Sylvatic**	**Peridomicile**	**Domicile**	**Sylvatic**	**Peridomicile**	**Domicile**
*Ps. coreodes*	46	43			3		
*P. guentheri*	6	5	1				
*T. breyeri*	3	2			1		
*T. delpontei*	18	15			3		
*T. eratyrusiformis*	4	4					
*T. garciabesi*	5	4	1				
*T. guasayana*	40	27	8	2	3		
*T. infestans*	129	23	60	21	1	17	7
*T. platensis*	29	27	1		1		
*T. sordida*	18	17					1
Total	298	167	71	23	12	17	8

Considering all samples, 261 were negative (87.58%) and 37 (12.41%) were positive for TrV. From the total of the TrV positive samples, 32% (n = 12) were from a sylvatic habitat, 46% (n = 17) from peridomicile habitat and 22% (n = 8) from a domicile habitat (Table 1). Although most species were found in the sylvatic habitat, we noticed that most of the samples (17) belong to the peridomicile.

All the samples that were naturally infected with TrV (n = 37) were used as occurrence data for the ENM procedure.

### Ecological niche modelling

#### Selection of environmental predictors

After a reduction in the number of Bioclim and AVHRR variables by the Jackknife procedure, a group of the most important environmental predictors was identified and is listed in Table A1 and A2 of the Additional file 1.

The identified environmental predictors were related with temperature derived statistics in both datasets. The MaxEnt procedure with the Bioclim dataset identified five environmental variables as best predictors including the following: temperature annual range, mean diurnal range and mean temperature of coldest quarter were the variables with the highest contribution to the mean model AUC, while temperature seasonality and annual mean temperature showed lower contribution. The MaxEnt procedure using the AVHRR dataset identified six environmental variables as best predictors, including minimum LST and minimum MIR, and LST and MIR amplitude of the annual cycle that showed the highest contribution to the mean model AUC; LST and MIR variance showed lower contribution to the final model.

The MaxEnt procedure showed that in terms of the final model AUC of the Bioclim dataset, the presence of TrV infected triatomines (with probability > 0.5) was associated with temperature annual range between 27 to 32°C, mean diurnal range between 14 to 16°C and mean temperature of the coldest quarter between 10 to 17°C (maximum at 17°C). In the case of the AVHRR dataset, presence of the TrV infected triatomines (with probability > 0.5) was associated with minimum MIR and LST values between 20.7 to 27°C, MIR annual amplitude values between 6 to 13°C, and LST annual amplitude values between 7.5 to 18.5°C. (see Figure A1-A7 in the Additional file 1).

#### Potential geographic distribution

**Figure 1:**
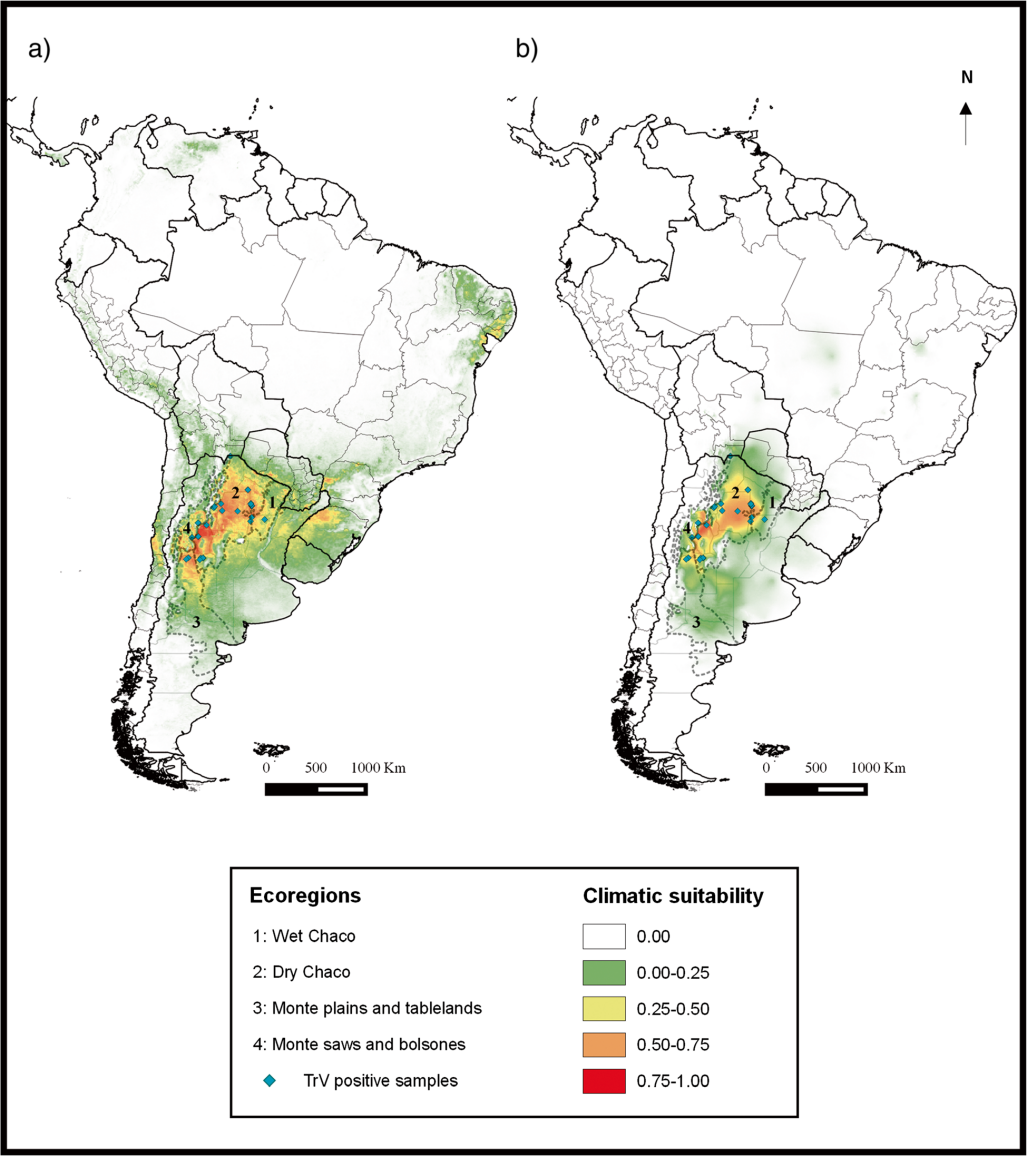
**Ecological niche models projected as potential geographic distribution for triatomine species positive to TrV (a) AVHRR (b) Bioclim.** Known occurrences of the species are shown as blue diamond. Dotted lines indicate the ecoregions.

The potential geographic distribution of triatomine species infected by TrV coincides with the Chaco (wet and dry) and the Monte (plains/tablelands and saws/bolsones) ecoregions [34], either modelled by AVHRR or Bioclim environmental datasets (Figure 1(a) and (b)).

Most predictions with occurrence probability > 0.5 modelled by both environmental datasets are mainly located in the dry Chaco, with remarkable “hot spots” in the provinces of La Rioja and Catamarca (Figure 1(a) and (b)).

The predictions provided by the modelling with the AVHRR dataset show a wider distribution of TrV in contrast with the Bioclim dataset, covering central and northern Argentina, mainly in the dry Chaco and in the northern part of the Monte plains and tablelands ecoregions. Other areas in neighbour countries such as Uruguay, Paraguay, Chile and Brazil are identified as areas of potential occurrence, although with very low probability (Figure 1(a)).

**Figure 2:**
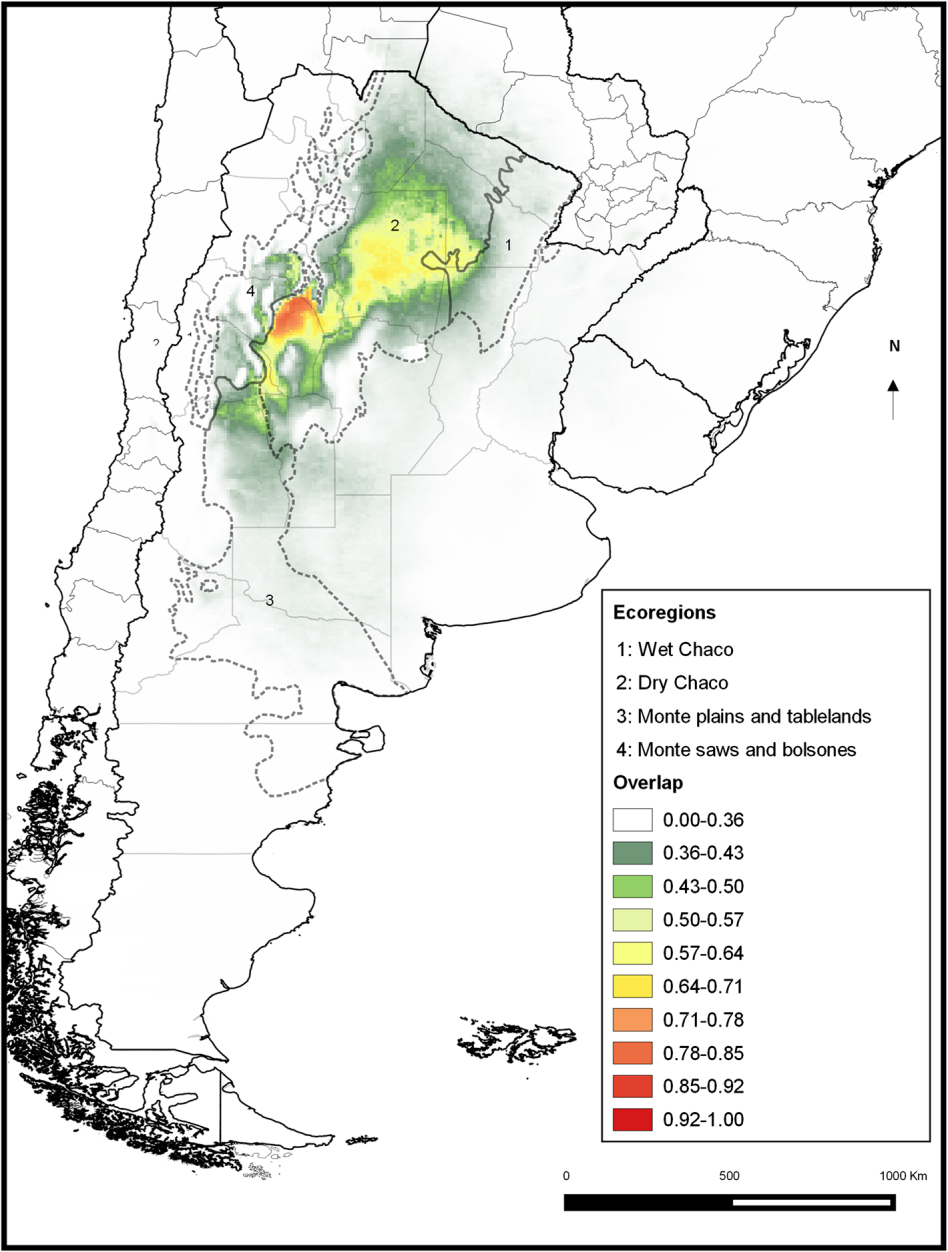
**Overlap between the potential distributions of triatomine species positive to TrV by the AVHRR- and Bioclim-based models.** Dotted lines indicate the ecoregions.

The area where both models overlap is associated with the dry Chaco region, with an area of high occurrence probability in the Llanos de La Rioja (Figure 2).

The average test AUC for the replicated runs was 0.989 and 0.985 for AVHRR and Bioclim variables, respectively, with the same standard deviation of 0.005.

## Discussion

There are few studies reporting TrV infection under field conditions [3,14,15] and only Marti *et al*. [3] reports the field records by localities of triatomine parasites and pathogens in Argentina.

From the seven triatomine species positive to TrV, six were found in the sylvatic habitat, one in the peridomicile habitat and two in the domicile habitat, indicating that TrV could be involved in different cycles (domicile, peridomicile and sylvatic), similar to the behaviour of *Trypanosoma cruzi* [35]. Being *T. infestans* the only species found infected with TrV in the three habitats, the sylvatic populations of this triatomine species could be responsible for the dispersion of the virus between different habitats by active dispersion [36].

According to Marti *et al*. [37], several triatomine species cohabit in the same microhabitat (e.g. *Ps. coreodes-T. sordida-T. platensis* and *T. infestans-T. platensis* in Furnaridae nests), therefore, it is likely that the infection by TrV may occur through horizontal transmission when the triatomines feed together [12], thus creating a virus circulation between different species in each habitat.

The ENM results using the two independent datasets of Bioclim and AVHRR show that the geographic distribution of TrV infection is strongly associated with the dry region of the Chaco ecoregion. Models based on the Bioclim variables of the WorldClim dataset are less sensitive to predict the full potential geographic distribution of the species, in contrast with models based on the remote sensor dataset (AVHRR) that predicted more accurately [38]. The potential distribution of triatomine species positive to TrV obtained is a first attempt to identify and characterize the climatically favourable areas in which the species could be infected naturally.

The resultant spatial distribution pattern for triatomines infected with TrV coincides mostly with the current potential geographic distribution of *T. infestans,* probably because most of the occurrence points belong to this species and/or because the geographic distributions of the other species are included within the *T. infestans* distribution. The amplitude and the minimum (coldest) temperatures were the main environmental determinants of distributional patterns, coinciding with previous findings that showed the capacity of this species to inhabit areas with broad ranges of temperatures [39]. According to Gorla [40], the geographic distribution of *T. infestans* using environmental variables recorded by earth observation satellites of the NOAA series was shown to be well described by a set of 7 environmental variables including statistics derived from air temperature, infrared radiation and vegetation index. In this work, using the same environmental variables, minimum MIR and minimum LST, and MIR and LST annual amplitudes turned out to be the variables that better described the potential distribution of TrV.

It is worth noting that Figure 1(a) shows an area in the northeast of Brazil that currently seems to harbour a small residual focus of *T. infestans*. Although this area shows a low probability of occurrence, the predictions could identify “hot spots” of these vector populations in neighbouring countries, where they have decreased after mostly successful triatomine control efforts.

## Conclusions

Although there are epidemiological studies of viruses transmitted by vectors of sanitary importance [41,42], this is the first study reporting the potential distribution of vectors infected naturally with one pathogenic virus that could be used as a potential biological control agent. Our results show that the conditions of the dry Chaco ecoregion in Argentina are favourable for the infection of one or more of the triatomine species analysed. PerhapsTrV was not found in the remaining three species (*T. eratyrusiforme*, *T. garciabesi* and *P. guentheri)*, either because this virus is not associated with these species yet, or because of the small sample size analysed of these three species.

We consider that our results suggest that the environmental variables provided by remote sensors (AVHRR) may be more suitable to use in Ecological Niche Modelling studies (at least in South America) because of the better precision instead of the meteorological environmental variables (Bioclim) that have widespread meteorological stations in this area.

This study is an additional step towards a complete geographic distributional summary of triatomines infected by TrV. However, to perform a more detailed and updated geographic distribution, this approach would require an increase in the number of points of occurrence and wider sampling of wild triatomine species, as well as sample collection in neighbour countries to the Argentina Gran Chaco, to corroborate the real presence of TrV.

## Additional file

Additional file 1: Table A1. Environmental variables from BIOCLIM dataset used in the modeling of distribution of triatomine species infected naturally with TrV. The variables marked (*) were selected as the best predictors by the MaxEnt Jackknife procedure. **Table A2.** Environmental variables from AVHRR dataset used in the modeling of distribution of triatomine species infected naturally with TrV. The variables marked (*) were selected as the best predictors by the MaxEnt Jackknife procedure. **Figure A1.** Response curve of distribution of triatomine species infected naturally with TrV to Mean Diurnal Range (Bio2). Units in the abscissa axis are x/10°C. **Figure A2.** Response curve of distribution of triatomine species infected naturally with TrV to Temperature Annual Range (Bio7). Units in the abscissa axis are x/10°C. **Figure A3.** Response curve of distribution of triatomine species infected naturally with TrV to Mean Temperature of Coldest Quarter (Bio11). Units in the abscissa axis are x/10°C. **Figure A4.** Response curve of distribution of triatomine species infected naturally with TrV to MIR annual amplitude (wd1003a1). Units in the abscissa axis are x/10°C. **Figure A5.** Response curve of distribution of triatomine species infected naturally with TrV to minimum MIR (wd1003mn). Units in the abscissa axis are (x/10)-273°C. **Figure A6.** Response curve of distribution of triatomine species infected naturally with TrV to LST annual amplitude (wd1007a1). Units in the abscissa axis are x/10°C. **Figure A7.** Response curve of distribution of triatomine species infected naturally with TrV to minimum LST (wd1007mn). Units in the abscissa axis are (x/10)-273°C.
