# Supplementary material for: Modelling the potential geographic distribution of triatomines infected by Triatoma virus in the southern cone of South America
Source: Parasit Vectors. 2015 Mar 12;8:153. doi: 10.1186/s13071-015-0761-1 (PMC4367828; doi:10.1186/s13071-015-0761-1)
Supplement: Additional file 1: Table A1. — Environmental variables from BIOCLIM dataset used in the modeling of distribution of triatomine species infected naturally with TrV. The variables marked (*) were selected as the best predictors by the MaxEnt Jackknife procedure. Table A2. Environmental variables from AVHRR dataset used in the modeling of distribution of triatomine species infected naturally with TrV. The variables marked (*) were selected as the best predictors by the MaxEnt Jackknife procedure. Figure A1. Response curve of distribution of triatomine species infected naturally with TrV to Mean Diurnal Range (Bio2). Units in the abscissa axis are x/10°C. Figure A2. Response curve of distribution of triatomine species infected naturally with TrV to Temperature Annual Range (Bio7). Units in the abscissa axis are x/10°C. Figure A3. Response curve of distribution of triatomine species infected naturally with TrV to Mean Temperature of Coldest Quarter (Bio11). Units in the abscissa axis are x/10°C. Figure A4. Response curve of distribution of triatomine species infected naturally with TrV to MIR annual amplitude (wd1003a1). Units in the abscissa axis are x/10°C. Figure A5. Response curve of distribution of triatomine species infected naturally with TrV to minimum MIR (wd1003mn). Units in the abscissa axis are (x/10)-273°C. Figure A6. Response curve of distribution of triatomine species infected naturally with TrV to LST annual amplitude (wd1007a1). Units in the abscissa axis are x/10°C. Figure A7. Response curve of distribution of triatomine species infected naturally with TrV to minimum LST (wd1007mn). Units in the abscissa axis are (x/10)-273°C. [file 13071_2015_761_MOESM1_ESM.docx]

ADDITIONAL FILE 1

**Table A1.** Environmental variables from BIOCLIM dataset used in the modeling of distribution of triatomine species infected naturally with TrV. The variables marked (*) were selected as the best predictors by the MaxEnt Jackknife procedure.

| **BIOCLIM** | **Units** |
| --- | --- |
| Annual Mean Temperature (Bio1) **(*)** | °C |
| Mean Diurnal Range (Bio2) **(*)** | °C |
| Isothermality [(Bio2/Bio7) * 100] (Bio3) | % |
| Temperature Seasonality (Bio4) **(*)** | °C |
| Max Temperature of Warmest Month (Bio5) | °C |
| Min Temperature of Coldest Month (Bio6) | °C |
| Temperature Annual Range (Bio7) **(*)** | °C |
| Mean Temperature of Wettest Quarter (Bio8) | °C |
| Mean Temperature of Driest Quarter (Bio9) | °C |
| Mean Temperature of Warmest Quarter (Bio10) | °C |
| Mean Temperature of Coldest Quarter (Bio11) **(*)** | °C |
| Annual Precipitation (Bio12) | mm |
| Precipitation of Wettest Month (Bio13) | mm |
| Precipitation of Driest Month (Bio14) | mm |
| Precipitation Seasonality (Coefficient of Variation) (Bio15) | mm |
| Precipitation of Wettest Quarter (Bio16) | mm |
| Precipitation of Driest Quarter (Bio17) | mm |
| Precipitation of Warmest Quarter (Bio18) | mm |
| Precipitation of Coldest Quarter (Bio19) | mm |

**Table A2.** Environmental variables from AVHRR dataset used in the modeling of distribution of triatomine species infected naturally with TrV. The variables marked (*) were selected as the best predictors by the MaxEnt Jackknife procedure.

| **AVHRR** | **Units** |
| --- | --- |
| MIR^a^ mean | °C |
| MIR^a^ annual amplitude (wd1003a1) **(*)** | °C |
| MIR^a^ bi-annual amplitude (wd1003a2) | °C |
| MIR^a^ tri-annual amplitude (wd1003a3) | °C |
| MIR^a^ % variance annual cycle (wd1003d1) | % |
| MIR^a^ % variance bi-annual cycle (wd1003d2) | % |
| MIR^a^ % variance tri-annual cycle (wd1003d3) | % |
| MIR^a^ % variance annual to tri-annual cycles (wd1003da) | % |
| minimum MIR^a^ (wd1003mn) **(*)** | °C |
| maximum MIR^a^ (wd1003mx) | °C |
| MIR^a^ phase 1 (wd1003p1) | months |
| MIR^a^ phase 2 (wd1003p2) | months |
| MIR^a^ phase 3 (wd1003p3) | months |
| MIR^a^ variance (wd1003vr) **(*)** | °C^2^ |
| LST^b^ mean (wd1007a0) | °C |
| LST^b^ annual amplitude (wd1007a1) **(*)** | °C |
| LST^b^ bi-annual amplitude (wd1007a2) | °C |
| LST^b^ tri-annual amplitude (wd1007a3) | °C |
| LST^b^ % of variance of original data described by annual cycle (wd1007d1) | % |
| LST^b^ % of variance of original data described by bi-annual cycle (wd1007d2) | % |
| LST^b^ % of variance of original data described by tri-annual cycle (wd1007d3) | % |
| LST^b^ % of variance of original data described by annual to tri-annual cycles (wd1007da) | % |
| minimum LST^b^ (wd1007mn) **(*)** | °C |
| maximum LST^b^ (wd1007mx) | °C |
| LST^b^ phase of annual cycle (wd1007p1) | months |
| LST^b^ phase of bi-annual cycle (wd1007p2) | months |
| LST^b^ phase of tri-annual cycle (wd1007p3) | months |
| LST^b^ variance (wd1007vr) **(*)** | °C^2^ |
| Mean NDVI^c^ (wd1014a0) | No units^d^ |
| NDVI^c^ annual amplitude (wd1014a1) | No units^d^ |
| NDVI^c^ bi-annual amplitude (wd1014a2) | No units^d^ |
| NDVI^c^ tri-annual amplitude (wd1014a3) | No units^d^ |
| NDVI^c^ % of variance of original data described by annual cycle (wd1014d1) | % |
| NDVI^c^ % of variance of original data described by bi-annual cycle (wd1014d2) | % |
| NDVI^c^ % of variance of original data described by tri-annual cycle (wd1014d3) | % |
| NDVI^c^ % of variance of original data described by annual to tri-annual cycles (wd1014da) | % |
| minimum NDVI^c^ (wd1014mn) | No units^d^ |
| maximum NDVI^c^ (wd1014mx) | No units^d^ |
| NDVI^c^ phase of annual cycle (wd1014p1) | months |
| NDVI^c^ phase of bi-annual cycle (wd1014p2) | months |
| NDVI^c^ phase of tri-annual cycle (wd1014p3) | months |
| NDVI^c^ variance (wd1014vr) | No units^d^ |

^a^ Middle Infrared Radiation

^b^ Land Surface Temperature

^c^ Normalized Difference Vegetation Index

^d^ No units because it is a ratio


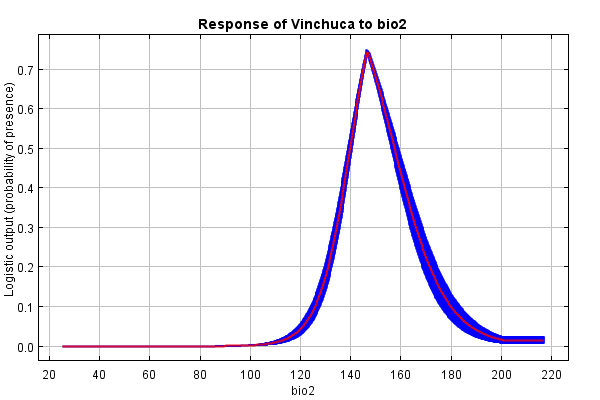


Fig A1. Response curve of distribution of triatomine species infected naturally with TrV to Mean Diurnal Range (Bio2). Units in the abscissa axis are x/10 ºC.


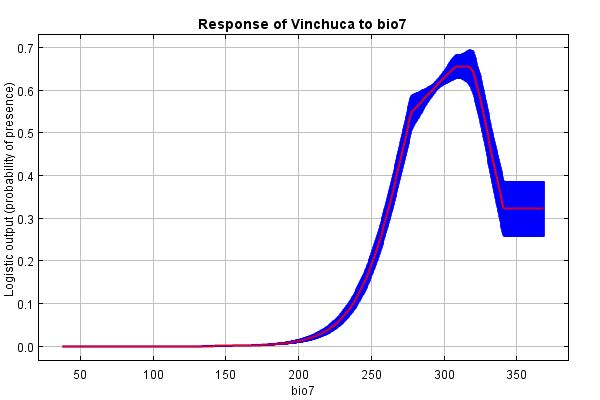


Fig A2. Response curve of distribution of triatomine species infected naturally with TrV to Temperature Annual Range (Bio7). Units in the abscissa axis are x/10 ºC.


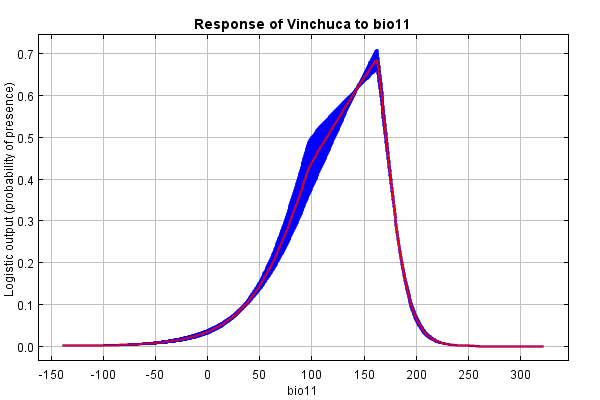


Fig A3. Response curve of distribution of triatomine species infected naturally with TrV to Mean Temperature of Coldest Quarter (Bio11). Units in the abscissa axis are x/10 ºC.


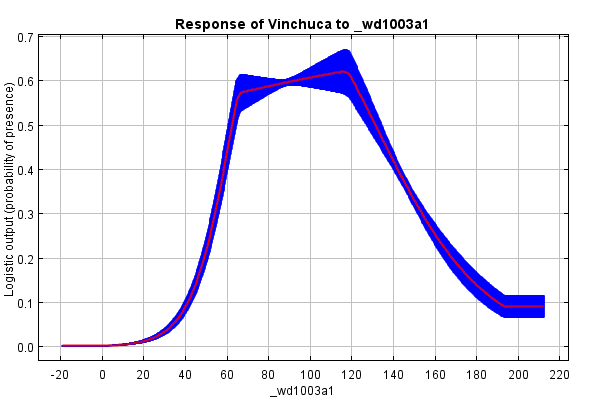


Fig A4. Response curve of distribution of triatomine species infected naturally with TrV to MIR annual amplitude (wd1003a1). Units in the abscissa axis are x/10 ºC.


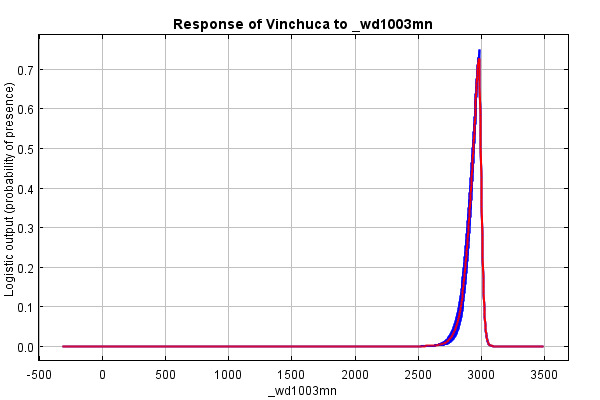


Fig A5. Response curve of distribution of triatomine species infected naturally with TrV to minimum MIR (wd1003mn). Units in the abscissa axis are (x/10)-273 ºC.


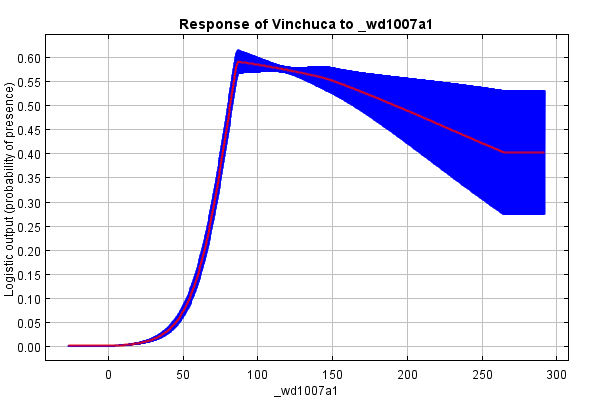


Fig A6. Response curve of distribution of triatomine species infected naturally with TrV to LST annual amplitude (wd1007a1). Units in the abscissa axis are x/10 ºC.


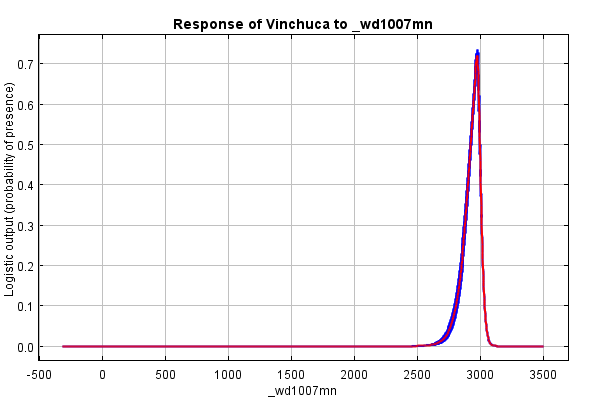


Fig A7. Response curve of distribution of triatomine species infected naturally with TrV to minimum LST (wd1007mn). Units in the abscissa axis are (x/10)-273 ºC.
